# Assessment of the Risk of Venous Thromboembolism in Overcrowded Shelters: A Geospatial and Statistical Analysis of the 2024 Noto Peninsula Earthquake

**DOI:** 10.7759/cureus.95558

**Published:** 2025-10-28

**Authors:** Hisao Nakai, Ryo Horiike, Tomoya Itatani, Mayu Tokuoka, Ayuka Maeda, Haruka Iida, Yoshie Aoki, Daisuke Toda, Masato Oe

**Affiliations:** 1 Nursing, University of Kochi, Kochi, JPN; 2 Nursing, Nara Medical University, Nara, JPN; 3 Nursing, University of Miyazaki, Miyazaki, JPN; 4 Nursing, Toyama Prefectural University, Toyama, JPN; 5 Nursing, Kanazawa Medical University, Kanazawa, JPN

**Keywords:** 2024 noto peninsula earthquake, deep vein thrombosis (dvt), economy class syndrome, evacuation shelters, geographical information system (gis), venous thromboembolism (vte)

## Abstract

Background: In recent years, Japan has frequently experienced natural disasters, including major earthquakes and extreme weather events linked to climate change, forcing many people into sheltered lives. These overcrowded environments are known to increase the risk of life-threatening venous thromboembolism (VTE). The 2024 Noto Peninsula Earthquake (NPE) similarly compelled numerous residents into prolonged shelter living.

Aim: This retrospective study aimed to indirectly assess VTE risk due to overcrowding in evacuation shelters using proxy indicators (occupancy rate, operational period) and identify spatial clusters of high-risk populations via geospatial and statistical analysis of open data from Nanao City, Ishikawa Prefecture, following the 2024 NPE, to inform disaster preparedness.

Methods: We analyzed open data on evacuation shelters in Nanao City, including location, capacity, and operational period, obtained from GitHub on June 3, 2024. VTE risk was assessed using three proxy indicators: occupancy rate, operational period, and the geographical concentration of overcrowding. The methods included descriptive statistics, geographic information system-based hotspot analysis, simple linear regression, and a sensitivity analysis of occupancy rates.

Results: Hotspot analysis identified a significant geographical cluster of the high-risk VTE population in central Nanao City (99% confidence level). Simple regression analysis showed a positive correlation between the cumulative number of evacuees and the operational period (R² = 0.48), though this trend was heavily influenced by a single significant outlier (R² = 0.35 after exclusion). The sensitivity analysis identified Ishizaki Elementary School (>90% occupancy) as the shelter with the most critical VTE risk due to overcrowding.

Conclusion: This study demonstrated that VTE risk from overcrowding was significantly concentrated in specific shelters, suggesting a potential for recurrence in future disasters under the current system. Municipalities should reconsider shelter placement and resource allocation to mitigate this risk. The quantitative, geographical, and open-data-based approach used here offers a low-cost, rapid, and widely applicable method for disaster preparedness.

## Introduction

Japan has experienced several catastrophic earthquakes, such as the Great Hanshin-Awaji Earthquake in 1995, the Great East Japan Earthquake in 2011, and the Kumamoto Earthquake in 2016, compelling large populations to live in evacuation shelters. In these shelters, many evacuees face conditions such as cramped living spaces, inadequate fluid intake, and limited access to shared toilets. Such circumstances have been reported to elevate the risk of venous thromboembolism (VTE) in a systematic review [1]. Given that deep vein thrombosis (DVT) can lead to a life-threatening pulmonary embolism (PE), the importance of early preventive intervention has been emphasized in evidence-based clinical practice guidelines [2]. The scale of this risk is illustrated by data from past disasters. For instance, following the 2011 Great East Japan Earthquake, DVT prevalence in Ishinomaki City surged to 250 times the normal rate [3], and national PE-related mortality exceeded 2,000 for the first time that year, highlighting the disaster's lethal consequences [4]. Furthermore, after the 2016 Kumamoto Earthquake, 9.5% of evacuees screened within 45 days were positive for DVT, with the older adults being disproportionately affected [5].

The 2024 Noto Peninsula Earthquake (NPE), which occurred on New Year's Day 2024, inflicted severe damage on the Noto Peninsula in Ishikawa Prefecture, Japan, compelling a peak of approximately 40,000 people to evacuate [6]. Nanao City, located near the center of the peninsula, suffered similarly extensive damage, including collapsed houses, fires, and landslides. Of the 263 fatalities in Ishikawa Prefecture, five occurred in Nanao City, where many residents were forced into shelter life [7]. A survey of a shelter for vulnerable individuals in Wajima City revealed the impact on evacuees: for the first seven days post-disaster, the facility was indiscriminately accessed not only by medically vulnerable people but also by residents of nearby damaged facilities and local community members, leading to severe overcrowding [8]. Furthermore, evacuation shelters in Wajima City faced issues such as deteriorating sanitary conditions, a lack of physical activity among older adults, the spread of infectious diseases, and delayed intervention by medical professionals like Disaster Medical Assistance Teams in isolated communities [9]. This overcrowding and poor sanitation can trigger secondary health complications, such as Economy Class Syndrome (ECS), attributable to DVT and VTE, and infectious diseases. According to health screenings conducted for disaster victims in the Okunoto region from January 8 to April 29, leg thrombosis was confirmed in 8.8% of individuals [10]. The onset of ECS is a critical issue that, in the worst cases, can lead to disaster-related death. A meta-analysis on DVT risk factors following earthquakes in Japan reported that the risk is elevated by a combination of immobility, stress, dehydration, and disruption of medical access [11]. In recent years, Japan has frequently experienced natural phenomena requiring emergency evacuation, not limited to major earthquakes but also including slow-moving and powerful tropical cyclones stemming from global warming, and localized torrential rains from linear precipitation bands [12,13]. Particularly in Japan, a nation grappling with a rapidly aging and declining population [14], assessing the risk of ECS in evacuation shelters and developing preventive strategies during non-disaster times represents a pressing public health challenge.

This study aimed to indirectly assess the risk of VTE using open data from Nanao City following the 2024 NPE. The condition known as ECS can cause DVT and subsequent life-threatening PE [15]. In this paper, these related conditions are collectively termed VTE, and we discuss the VTE risk associated with overcrowding in evacuation shelters. The findings presented herein can provide evidence for planning the allocation of human and material resources for preventive interventions in future disaster management. This research has the potential to contribute to policies aimed at safeguarding the lives and health of as many evacuees as possible. Furthermore, these findings are relevant not only to Japan but also to other countries that have recently experienced climate change-related disasters and are projected to face significant population aging.

## Materials and methods

Study design and data source

This is an observational ecological study utilizing an open dataset, which was published on GitHub [16] by a volunteer based on publicly available data from Nanao City. The unit of analysis was the evacuation shelter (n = 32) at the facility level, not individual evacuees, within the setting of Nanao City, following the NPE on January 1, 2024. The analysis utilized a single dataset compiled by a volunteer (username: raokiey) based on official information from Nanao City.

Data source and variables

**Figure 1:**
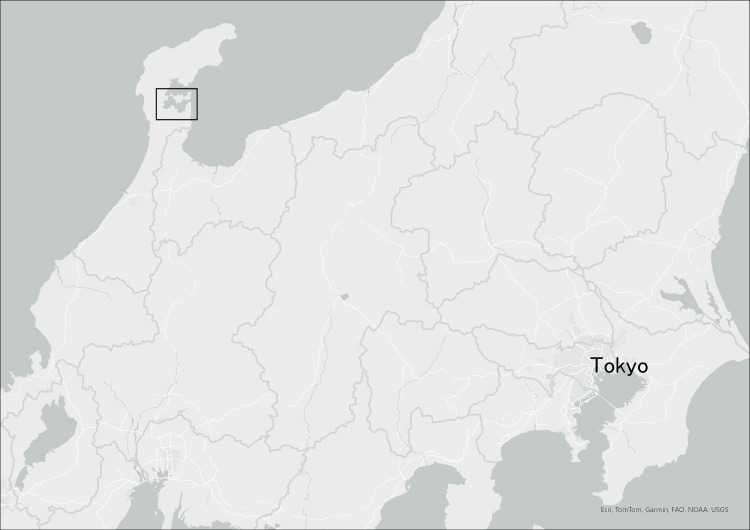
The location of Nanao City in Ishikawa Prefecture, Japan (e.g., Nanao City Hall, centered at 37.04° N, 136.96° E)

For this study, the data were downloaded from the "raokiey/R06-Noto-Peninsula-EQ-open-shelter-Nanao" repository on GitHub [16] in GeoJSON format on June 3, 2024. To ensure reproducibility, this specific version was utilized. While the authors of this paper were not involved in the primary data collection, we performed a sample cross-check of key data points (e.g., shelter names and addresses) against the official city announcements to verify consistency. The analysis utilized the following variables from the dataset: shelter name, address, geographic coordinates (latitude and longitude), and operational status (total cumulative number of evacuees, operational period, and shelter capacity). This dataset is provided under the Creative Commons Attribution 4.0 International license, permitting its use for research purposes. The location of Nanao City in Ishikawa Prefecture, Japan, is shown in Figure 1.

Analytical methods

In this study, we assessed the risk of VTE attributable to evacuee overcrowding in evacuation shelters in Nanao City, Ishikawa Prefecture, during the 2024 NPE, using publicly available open data. For the VTE risk assessment, we defined the following proxy indicators. First, we used the shelter occupancy rate as an indicator of overcrowding. This is because overcrowding in shelters, due to a lack of space, can force individuals to maintain static postures for extended periods and lead to a lack of physical activity, constituting a physical environment with a high risk for VTE. Second, we utilized the operational period of the shelters, as prolonged stays in such a high-risk environment can cumulatively increase VTE risk. Third, we employed shelter overcrowding density, determined via hotspot analysis, as an indicator of the geographical concentration of VTE high-risk populations within the region.

First, descriptive statistics were calculated for the total cumulative number of evacuees and the operational period of the shelters to understand their distributions. Using a geographic information system (GIS), the geographic coordinates of the evacuation shelters were plotted on a map. A hotspot analysis was then performed using spatial analysis tools, referencing the distance between shelters and the number of evacuees, to identify hotspots at 90%, 95%, and 99% confidence levels. Next, to quantitatively evaluate the relationship between the total cumulative number of evacuees and the operational period, a simple linear regression analysis was conducted, with the former as the explanatory variable and the latter as the dependent variable. Simple linear regression was employed to explore and quantify the potential linear relationship between the continuous variables of shelter operational period and cumulative number of evacuees. The key assumptions for simple linear regression were considered. Linearity was visually assessed using scatter plots. Independence of errors was assumed, as each shelter represented a distinct observation. While homoscedasticity and normality of errors were not formally tested due to the exploratory nature of the analysis, the small sample size (n = 32), and the presence of an outlier, these aspects were carefully considered during the interpretation of the results. Initially, the relationship was visually inspected using a scatter plot of the entire dataset (n = 32). A regression line was fitted, and an outlier that deviated significantly from this line was identified. To assess the impact of this outlier, the regression analysis was performed in two stages: Model 1, using the full dataset (n = 32, including the outlier), and Model 2, using the dataset with the outlier excluded (n = 31). The strength of the linear relationship in each model was then compared using the coefficient of determination (R²). Furthermore, referencing prior studies that link high shelter occupancy rates to the onset of ECS [17,18], we conducted a sensitivity analysis by varying the threshold for shelter occupancy rate from 50% to 100%.

All statistical analyses were performed using Microsoft Excel (Office 365; Microsoft, Redmond, WA, USA), and the level of statistical significance was set at p < 0.05. Spatial analyses were conducted using ArcGIS Pro (Ver 3.2.1; Esri, Redlands, CA, USA).

Ethical consideration

This study utilized only anonymized, open-access data, ensuring that no individuals could be identified. All applicable terms of use and laws were strictly adhered to.

## Results

Overview of evacuation centers in Nanao City

Following the 2024 NPE, 32 (50.0%) of the 64 designated evacuation centers in Nanao City were utilized. The median operational period for these centers was 50.5 days (interquartile range (IQR), 19.3-57.0), and the median cumulative number of evacuees was 878.5 (IQR, 555.0-2,924.0). The length of these operational periods and the scale of the evacuee population form the essential background for assessing the risk of VTE in this study. The distribution of the evacuation center operational periods and the cumulative number of evacuees is presented in Table 1.

**Table 1:** Overview of evacuation centers in Nanao City Median (interquartile range, IQR) was used to represent central tendency and dispersion due to the observed asymmetry in data distribution (as indicated by skewness and kurtosis values), which provides a more appropriate description than mean (standard deviation). Minimum and maximum values are included to illustrate the full range of the data and the presence of potential outliers

Items	Median (IQR)	Minimum	Maximum	Skewness	Kurtosis
Shelter operation period (days)	50.5 (19.3−57.0）	1	120	0.39	2.52
Cumulative number of evacuees (people)	878.5 (555.0−2,924.0)	13	12,350	-0.17	8.17

Geographic concentration of the high-risk VTE population: hotspot analysis

**Figure 2:**
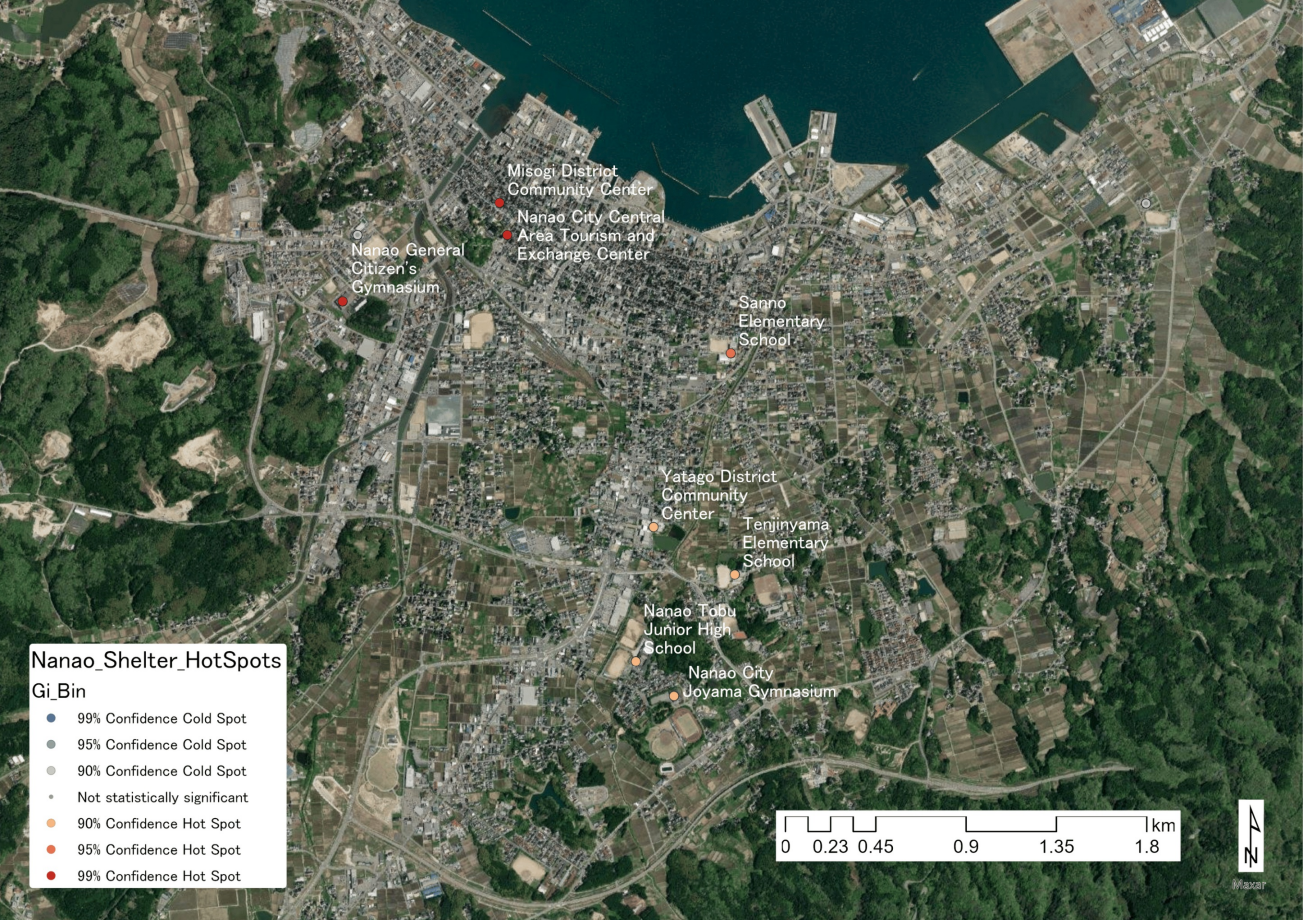
Results of the hotspot analysis evaluating the geographic concentration of the high-risk VTE population VTE: venous thromboembolism

The hotspot analysis identified statistically significant hotspots. The most significant cluster, at a 99% confidence level, was formed in an area encompassing the Nanao City Central Area Tourism and Exchange Center, the Misogi District Community Center, and the Nanao General Citizens Gymnasium. A hotspot at the 95% confidence level was detected at Sanno Elementary School, while hotspots at the 90% confidence level were identified at the Nanao City Ooyama Gymnasium, Nanao Tobu Junior High School, Tenjinyama Elementary School, and Yatago District Community Center. The location of each evacuation center and the results of the hotspot analysis are illustrated in Figure 2.

Relationship between operational period and cumulative number of evacuees

**Figure 3:**
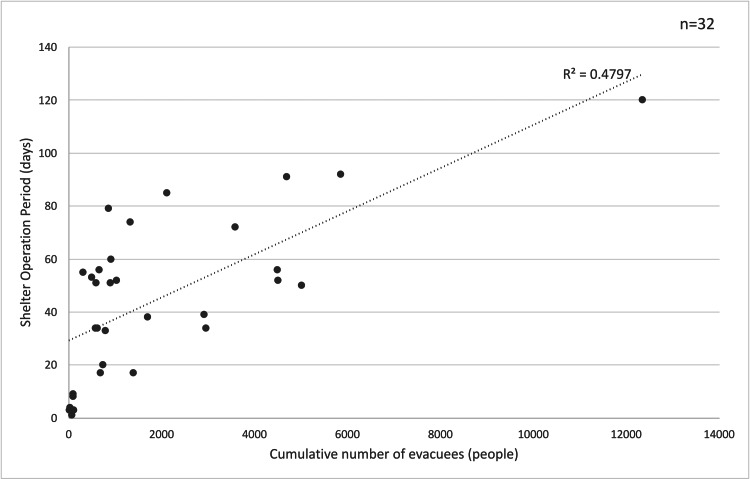
Relationship between the operational period and the cumulative number of evacuees (Model 1: all data, n = 32) p < 0.001, β = 0.008

**Figure 4:**
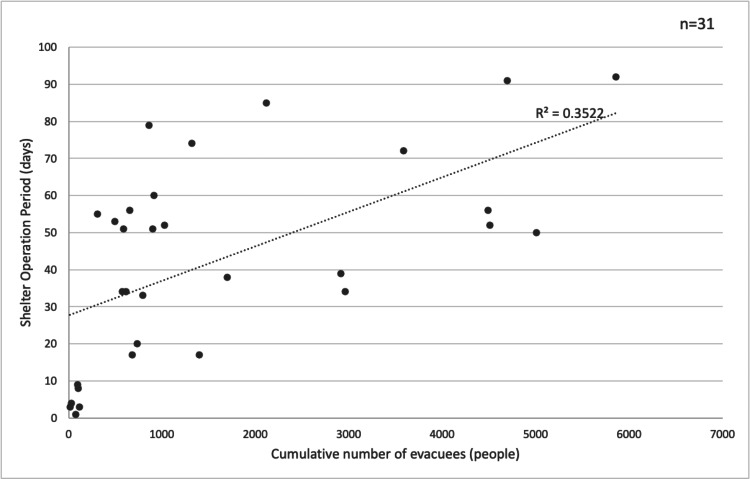
Relationship between the operational period and the cumulative number of evacuees (Model 2: outlier excluded, n = 31) p < 0.001, β = 0.009

A simple linear regression analysis was conducted to evaluate the effect of the operational period on the cumulative number of evacuees. A scatter plot of the entire dataset (n = 32) revealed that the Yatago District Community Center was a significant outlier, with 12,350 cumulative evacuees and an operational period of 120 days. To account for the influence of this outlier, a second model, Model 2 (n = 31), was created by excluding the Yatago District Community Center. The results of the simple regression analysis, with and without the outlier, are presented in Figures 3, 4. Model 1, which included all data, showed a coefficient of determination (R²) of 0.4797 and a correlation coefficient (r) of 0.69. In contrast, Model 2, which excluded the outlier, had an R² value of 0.3522 and an r value of 0.59. This suggests that the R² value of Model 1 may have been overestimated due to the influence of this significant outlier.

Identifying evacuation centers with high VTE risk due to overcrowding

**Figure 5:**
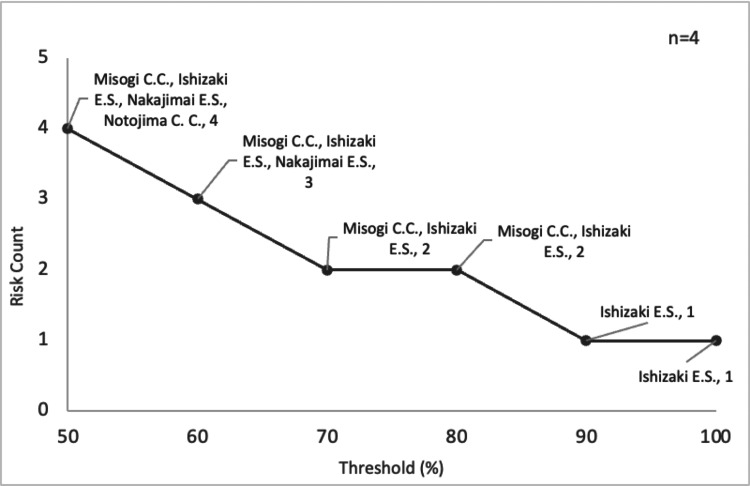
Fluctuation in the number of at-risk centers with changes in the evacuation center occupancy rate threshold To improve the readability of facility names in the figure, "Community Center" and "Elementary School" have been abbreviated as "C.C." and "E.S.," respectively

To assess the robustness of identifying high-risk shelters to varying definitions of overcrowding, a threshold sensitivity analysis was conducted. This was crucial because the specific threshold for occupancy rate, used as a proxy for VTE risk, can significantly influence which shelters are identified as “high-risk.” In this sensitivity analysis, the occupancy rate threshold was varied from 50% to 100%, and the number of centers exceeding each threshold was evaluated. According to this analysis, four centers exceeded a 50% occupancy rate. When the threshold was raised to 60%, the number of at-risk centers decreased to three. Ishizaki Elementary School was the only facility that surpassed the 90% and 100% occupancy thresholds. The results of this sensitivity analysis are shown in Figure 5.

## Discussion

In this study, we analyzed the VTE risk for evacuees based on data from evacuation centers in Nanao City during the 2024 NPE. Three aspects were used as proxy indicators for this risk: geographic concentration, long-term exposure to a high-risk environment, and overcrowding. The results revealed a trend of elevated VTE risk at certain key evacuation centers that were both overcrowded and operational for extended periods, leading to the formation of hotspots in central Nanao City (Figure 2). In particular, the findings suggest that Ishizaki Elementary School, with an occupancy rate exceeding 90%, was exposed to a potentially critical level of VTE risk.

The GIS-based hotspot analysis revealed that the most significant hotspot was located in central Nanao City, around the Nanao City Central Area Tourism and Exchange Center. Considering that the evacuation center environment itself elevates VTE risk [2], this suggests that the area may represent not just a concentration of evacuees but also the geographic epicenter of a high-risk state for VTE development. If the current layout of evacuation centers in Nanao City remains as it was during the 2024 NPE, it is presumed that a future hazard of similar magnitude-such as a major earthquake or torrential rains requiring evacuation-would lead to a similar aggregation of evacuees, consequently increasing VTE risk again. This highlights the need to reconsider the number and placement of evacuation centers and to establish a scheme that allows for the early implementation of VTE prevention strategies.

In the simple regression analysis, the positive correlation between the cumulative number of evacuees and the operational period indicates a tendency for centers with larger populations to remain open longer. The Yatago District Community Center, a significant outlier, represents a case where an extremely large number of evacuees resided for a prolonged period. This suggests that, for the reasons previously mentioned [2], their exposure to VTE risk was particularly severe. The decrease in the coefficient of determination after excluding this outlier indicates that the overall trend is weaker without such an extreme case. Model 2 (R² = 0.35), which excluded the outlier, suggests that for centers other than Yatago, the operational period, and thus the duration of VTE risk exposure, is not determined solely by the cumulative number of evacuees. The remaining 65% of the variance may be influenced by other factors identified in previous research [19], such as the designation of centers as key multifunctional evacuation shelters, the status of infrastructure recovery, and administrative decisions on shelter operations. This implies that while most shelters were closed or consolidated within a certain timeframe, the Yatago District Community Center alone was compelled to operate on a unique, large-scale, and ultra-long-term basis, which significantly heightened VTE risk. To contextualize this, a study predicting shelter congestion in Tokyo cited factors such as dehydration, power outages, lifeline disruptions, and a mismatch between the actual population and shelter plans as causes for severe overcrowding [20]. Therefore, analyzing why the Yatago District Community Center became such an outlier, taking into account the local population and specific environmental conditions, is crucial for identifying and addressing future challenges in evacuation center management.

The sensitivity analysis based on shelter occupancy rates indicates that Ishizaki Elementary School, which experienced extreme overcrowding with an occupancy rate of over 90%, can be considered the shelter with the most critical VTE risk identified in this study. Its risk level is substantially different from that of shelters with 50% or 60% occupancy rates. This finding suggests the need to explore countermeasures for the anticipated overcrowding at this school, such as pre-designating multiple alternative shelters in the vicinity and decentralizing resource allocation.

Given the recent frequency of disasters in Japan, caused not only by major earthquakes but also by climate change-related events such as torrential rains, heavy snow, and heatwaves [21,22], the estimates from this study provide critical evidence for the need to proactively reassess evacuation center placement from a VTE prevention perspective. The VTE risk assessment methodology used in this study, which relies on open data, is advantageous due to its low cost and capacity for rapid evaluation. This approach provides implications for reviewing shelter placement and resource allocation in municipalities like Nanao City, and its low-cost and rapid evaluation capabilities could potentially contribute to the formulation and review of future regional disaster prevention plans. It represents a highly versatile approach for protecting populations from the health consequences of VTE during the large-scale disasters that are occurring with increasing frequency worldwide. As such, this method is readily applicable to other regions.

This study has several limitations. First, the study only indirectly assessed VTE risk through proxy indicators. Although previous research suggests a correlation between factors like shelter occupancy rates or operational periods and VTE risk, these are merely surrogate measures and do not represent the actual VTE incidence rate. Second, our analysis was restricted to variables available in open data. Consequently, individual-level risk factors that significantly influence VTE development, such as evacuee demographics, medical history, fluid intake, and personal space per capita, were not considered. Therefore, an assessment of high VTE risk due to overcrowding in this study does not definitively prove an actual risk of VTE incidence. Third, the estimations are based solely on cumulative evacuee numbers, total shelter operational periods, and location data. This approach does not account for the dynamic movement of people. For instance, the data may include individuals who, due to factors like power and water outages, stayed in shelters only during the day while returning to cars or homes at night (or vice versa). Furthermore, in areas identified as hotspots, frequent movement of people between shelters may have occurred. Our analysis, however, could not incorporate these time-based population dynamics.

## Conclusions

Using open data from the 2024 NPE, this study demonstrated that the risk of VTE due to evacuee overcrowding was significantly concentrated in specific regions and shelters. This finding on risk concentration can serve as a crucial guideline for protecting lives and health in future disasters. In light of the severity of VTE, we recommend that municipal governments re-examine their policies based on the characteristics of the hotspots and high-risk shelters identified herein. This policy review should include measures to prevent an excessive burden on specific shelters, such as decentralizing their placement and formulating robust plans that encourage residents to evacuate early. This study's approach-spatial statistical analysis using open GIS data for VTE risk during disasters using only widely available open data-has the potential to become a powerful, low-cost, and rapid method for protecting lives in preparation for future disasters.
